# Supplementary figures and images for: Effects of afforestation on water resource variations in the Inner Mongolian Plateau
Source: PeerJ. 2019 Nov 6;7:e7525. doi: 10.7717/peerj.7525 (PMC6842297; doi:10.7717/peerj.7525)

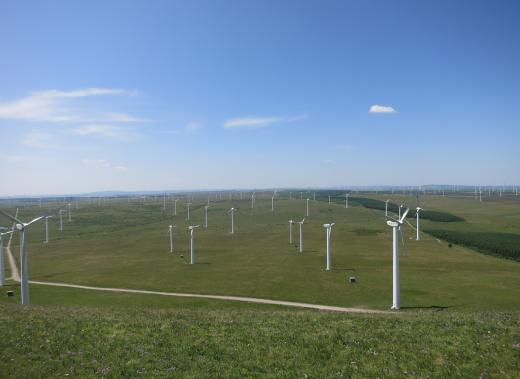

Supplement: Figure S2A [file peerj-07-7525-s002.jpg]

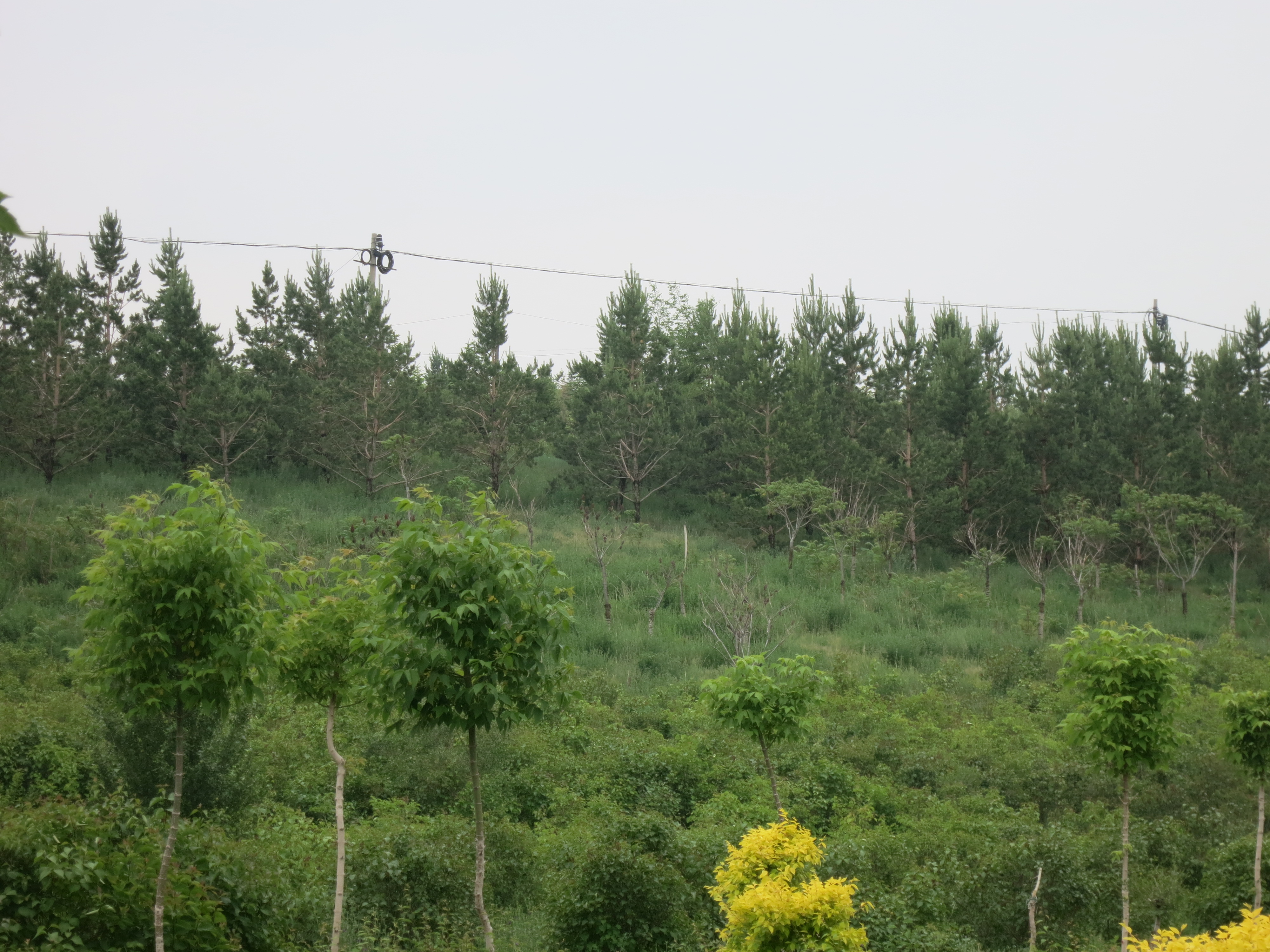

Supplement: Figure S2B [file peerj-07-7525-s003.jpg]
